# Supplementary material for: Is it really “panic buying”? Public perceptions and experiences of extra buying at the onset of the COVID-19 pandemic
Source: PLoS One. 2022 Feb 25;17(2):e0264618. doi: 10.1371/journal.pone.0264618 (PMC8880905; doi:10.1371/journal.pone.0264618)
Supplement: S1 File — (DOCX) [file pone.0264618.s001.docx]

**Supporting Information 1.** List of interview questions

**Demographics**

· Age

· Location

· Gender

· Employment

**Intro questions**

· Where do you live? How long have you been living in X place for?

· Do you work? Full time/part time?

· Has the pandemic affected you in any way?

· Can you work from home?

· Did you or anyone you know show symptoms?

· Did you or anyone you know self-isolate before the lockdown (UK)?

· Did you / Are you self-isolating now?

**Personal reactions, decisions, and sources of influence**

· How did you **react** when you first heard about the pandemic in general (China)?

· How did you **react** when you first heard about the pandemic in your country/local area?

o How did you feel?

o What were your first decisions?

· Did you **prepare**/**are you preparing** in any way to protect against the outbreak?

o How? What did you do?

o Why did you act in this way?

o If not, why not?

· Do/did you worry that there might be a **shortage** of food or other goods? Why yes/no?

· Did you **visit supermarkets** or other shops to acquire supplies?

o If yes, where did you go/what did you buy?

o Is that **more** than what you would usually buy?

o If yes, why did you **buy more than usual**? Why yes/not?

o If not, why not?

o Have you visited the supermarket or ordered food and supplies more times than usual? What is the main reason for your buying?

o Did you witness any empty selves?

§ In person?

§ On (social) media?

§ If yes, how did it make you feel?

· Did the **official announcements** have an effect on your preparation?

o Why yes/no? How?

o Did you believe the government statements that there is enough food or that you don’t need to panic?

· Did **social media** have any effects on your preparation?

o Did you come across any such posts? Where?

o Why yes/why not?

o Who were these posts by? (e.g. people you know?)

o How about your shopping habits today? Have they changed, or do they remain the same?

**Other people’s actions**

· Did other people’s behaviour have any effect on your preparations?

· How do you think that **other people react(ed)** to the virus?

o How do you think they felt psychologically when the pandemic first started?

o How did they feel when the pandemic hit the UK?

· Have others prepared?

o What have you witnessed? Could you please give an example? How did other people behave?

· Where did you witness it?

o Do you think that people bought more supplies than usual?

o Why yes/not?

o Where did you come across such instances? Social media? TV?

o What are the factors that can affect people’s behaviour? Why might they be influenced to behave in specific ways? (Media, other people’s behaviour etc).

· How do you think that people react in general when such incidents occur?

o Do you think that people act competitively? Why yes/no?

o Do they express solidarity? Why yes/no?

o Do images of empty selves affect how people react?

o How? In what way?

o Why?

· Has your view of the pandemic changed since you first heard about it? Yes - why, what made it change?

· Has your behaviour changed since the first week (since you first heard about it)? Why, what made it change?
