# Supplementary material for: Is it really “panic buying”? Public perceptions and experiences of extra buying at the onset of the COVID-19 pandemic
Source: PLoS One. 2022 Feb 25;17(2):e0264618. doi: 10.1371/journal.pone.0264618 (PMC8880905; doi:10.1371/journal.pone.0264618)
Supplement: Attachment — Submitted filename: Response to reviewers.docx [file pone.0264618.s002.docx]

We would like to thank the editor and the reviewers for their positive comments on our paper as well as for the opportunity to resubmit. In this letter, we address all comments raised. Our responses appear in red below the comments.

Kind regards,

The authors

1. Please ensure that your manuscript meets PLOS ONE's style requirements, including those for file naming. The PLOS ONE style templates can be found at

<https://journals.plos.org/plosone/s/file?id=wjVg/PLOSOne_formatting_sample_main_body.pdf> and

<https://journals.plos.org/plosone/s/file?id=ba62/PLOSOne_formatting_sample_title_authors_affiliations.pdf>

We have added information in the authors’ affiliations. Also, we have removed funding information from the acknowledgments section and have added a ‘Supporting Information’ section at the end of the manuscript. Supporting information documents as well as the manuscripts have been named accordingly. We have uploaded a manuscript with track changes (in red) and an identical manuscript without track changes.

2. Please amend your current ethics statement to address the following concerns:

a) Did participants provide their written or verbal informed consent to participate in this study?

b) If consent was verbal, please explain i) why written consent was not obtained, ii) how you documented participant consent, and iii) whether the ethics committees/IRB approved this consent procedure.

We have now addressed these comments, which were also shared by Reviewer 2. Please see our response to Reviewer 2 below regarding the text that we have added in our methods section in which we explain the procedure we followed to obtain participants’ consent. In short, consent was verbal after participants had read both the participant information sheet and consent form. Consent was obtained by participants stating it in the recording before the interviews commenced (hence it has been recorded). This form of consent was approved by the ethics committee due to COVID restrictions.

3. Thank you for stating the following financial disclosure:

“The research presented here was supported by a QR seed grant by the School of Psychology and Life Sciences at Canterbury Christ Church University awarded to Evangelos Ntontis, and by a UKRI grant awarded to John Drury, Clifford Stott, Stephen Reicher, Fergus Neville and Evangelos Ntontis (ES/V005383/1).”

Please state what role the funders took in the study. If the funders had no role, please state: "The funders had no role in study design, data collection and analysis, decision to publish, or preparation of the manuscript."

If this statement is not correct you must amend it as needed.

Please include this amended Role of Funder statement in your cover letter; we will change the online submission form on your behalf.

We have amended the financial disclosure, which now reads as:

*The research presented here was supported by a QR seed grant by the School of Psychology and Life Sciences at Canterbury Christ Church University awarded to Evangelos Ntontis, and by a UKRI grant awarded to John Drury, Clifford Stott, Stephen Reicher, Fergus Neville and Evangelos Ntontis (ES/V005383/1). The funders had no role in study design, data collection and analysis, decision to publish, or preparation of the manuscript.*

4. Thank you for stating the following in the Acknowledgments Section of your manuscript:

“The research presented here was supported by a QR seed grant by the School of Psychology and Life Sciences at Canterbury Christ Church University awarded to Evangelos Ntontis, and by a UKRI grant awarded to John Drury, Clifford Stott, Stephen Reicher, Fergus Neville and Evangelos Ntontis (ES/V005383/1).”

We note that you have provided additional information within the Acknowledgements Section that is not currently declared in your Funding Statement. Please note that funding information should not appear in the Acknowledgments section or other areas of your manuscript. We will only publish funding information present in the Funding Statement section of the online submission form.

Please remove any funding-related text from the manuscript and let us know how you would like to update your Funding Statement. Currently, your Funding Statement reads as follows:

“The research presented here was supported by a QR seed grant by the School of Psychology and Life Sciences at Canterbury Christ Church University awarded to Evangelos Ntontis, and by a UKRI grant awarded to John Drury, Clifford Stott, Stephen Reicher, Fergus Neville and Evangelos Ntontis (ES/V005383/1).”

Please include your amended statements within your cover letter; we will change the online submission form on your behalf.

We have now removed the financial statement from the main manuscript. The financial/funding statement that we would like to include is the following:

*The research presented here was supported by a QR seed grant by the School of Psychology and Life Sciences at Canterbury Christ Church University awarded to Evangelos Ntontis, and by a UKRI grant awarded to John Drury, Clifford Stott, Stephen Reicher, Fergus Neville and Evangelos Ntontis (ES/V005383/1). The funders had no role in study design, data collection and analysis, decision to publish, or preparation of the manuscript.*

5. Please review your reference list to ensure that it is complete and correct. If you have cited papers that have been retracted, please include the rationale for doing so in the manuscript text, or remove these references and replace them with relevant current references. Any changes to the reference list should be mentioned in the rebuttal letter that accompanies your revised manuscript. If you need to cite a retracted article, indicate the article’s retracted status in the References list and also include a citation and full reference for the retraction notice.

We have reviewed our reference list and verify that details are accurate, links are working, and no papers have been retracted since our paper was submitted. Thus, no changes to the reference list have been made.

**Comments from Reviewer 1**

Reviewer #1: The study is quite interesting and also it is well-written article. I have thoroughly gone through the whole article and found it to be quite interesting paper with good policy implication. Authors have worked alot on all the sections of the paper and thus, I strongly support this article for publication without any further changes.

Thank you for your kind words and suggestion to accept the paper as it is.

**Comments from Reviewer 2**

Reviewer #2: I would first like to extend appreciation for your work. Interesting set of thematic analyses. Please find below minor comments/questions:

1. Consent statements have not been included in your manuscript. While it is good that you've included the ethical statement, it would also be important to include the consent statement as well.

We have included the following statement in the method section: *“Before the interviews commenced, participants had to provide the interviewers with their consent. Since the interviews were carried out online or while observing social distancing measures, a consent form was sent to participants in advance electronically together with a participant information sheet which described the aims of the project. Participants then had to confirm verbally that they read and understood the information sheet, that their participation was voluntary and that they could withdraw at any point without the need to provide a reason, that they agreed to the interview being audio recorded, that their information would be kept strictly confidential in line with the lead author’s University Research Privacy Notice, and that they agreed to take part in the project overall.”*

2. Can the authors clearly state their exclusion criteria? (Before I read your "limitations" segment, I assumed that you deliberately focused on a specific demographic)

Thank you for this observation. Our inclusion criteria are discussed in the ‘participants, recruitment, and interview questions’ section, where we state that ‘*Our only inclusion criteria were that participants were over 18 years old and resided in the United Kingdom at the onset and through the early course of the pandemic*’. However, we also added the following sentences which make our sampling strategy more explicit: ‘*We used opportunity sampling and participants were recruited through personal contacts, snowballing, and social media (Facebook, Twitter) on the basis of their willingness to participate in our study. Thus, the sample is not representative of the UK population*’

3. I believe you must address the sample size as a limitation maybe, to note that it is in fact to small to be used for generalization of the topic under discussion.

We have added a few lines in our discussion where we highlight sample size limitations as well: “*Third, our sample was relatively small (but nevertheless typical for semi-structured, qualitative interview studies), young, and not representative in terms of age, socioeconomic status, or geographical location. These factors might have affected participants’ risk perceptions and risk tolerance, affecting the findings. Based on the aforementioned limitations, future research should collect data at the time that stockpiling behaviours are observed and from larger and representative samples*.”
